# Domestic sewage as a sustainable freshwater substitute for enhanced anaerobic digestion of lignocellulosic biomass

**DOI:** 10.1038/s41598-024-83546-6

**Published:** 2024-12-30

**Authors:** TG Induchoodan, Nimitha Choran, Ajay S. Kalamdhad

**Affiliations:** Department of Civil Engineering, Indian Institute of Technology Guwahati, Guwahati, Assam 781039 India

**Keywords:** Biogas, Domestic wastewater, Lignin degradation, Biochemical methane potential, Water hyacinth, *Hydrilla verticillata*, Biogas, Bioenergy, Biofuels

## Abstract

Biochemical methane potential tests using water hyacinth (WH), pretreated water hyacinth (PWH), and *Hydrilla verticillata* (HV) as substrates using sewage media were explored. This study replaced the freshwater required to prepare the slurry for AD of organic solid waste with domestic sewage. Cow dung was used as the inoculum. WH (241.5 mL CH_4_/g VS_added_), PWH (200.5 mL CH_4_/g VS_added_), and HV (212 mL CH_4_/g VS_added_) produced significant amounts of methane in the sewage medium. 16S-rRNA analysis showed that, in sewage, ~ 85% of the microbes were hydrolytic bacteria, and 7% were methanogens. This abundant quantity of hydrolytic microbes from sewage accelerated lignin degradation, achieving 28.32% and 38.34% degradation for WH and HV, respectively, within 14 days. Field emission-scanning electron microscopy images visually confirmed the enhanced substrate degradation in the presence of sewage. The net energy produced from the AD of WH and HV was significant (4664 J/g VS_added_ and 4109 J/g VS_added_), but for PWH, it was negative, indicating that using sewage medium may be better than costly pretreatment techniques. This study demonstrated the potential of using sewage as an alternative to freshwater in AD, offering a sustainable solution for freshwater conservation and the possible utilisation of sewage for improved methane production, especially for substrates with lignin that are difficult to degrade.

## Introduction

Anaerobic digestion (AD) involves the microbial degradation of organic matter in the absence of oxygen, resulting in the production of CH_4_, CO_2_, and some other trace gases and is collectively known as biogas^1^. Despite the long degradation period, AD generates a significantly low volume of sludge, which, along with biogas production, makes it an appealing choice for waste management and energy production. The recent focus on alternative energy sources has pushed AD to the limelight. The government of India has launched various schemes to attract private and public entities to install AD plants^2^. The adoption of AD faces several challenges, such as a lack of financing mechanisms, competition from other fuels, and feedstock variation^3^. Despite this, through the collective efforts of various stakeholders, such as public, private, and government entities, numerous AD plants have been installed and started operating in the past few decades. The smooth operation of an AD plant depends on various factors such as the steady supply of substrate, the presence of skilled labourers to maintain the process stability, the facility to manage the sludge and digestate produced and economic feasibility.

In recent years, studies have shown that maintaining an optimum moisture content is crucial for effectively treating organic waste using AD^4,5^. The optimum moisture content is achieved by adding freshwater while preparing the substrate slurry. The substrate slurry, prepared by shredding and grinding the substrate and then mixing it with an adequate quantity of freshwater, is essential for the operational ease of pumping the feed to the reactor. Low moisture content limits the microbial access to substrates and encounter difficulties in agitation and homogenisation, leading to an increase in localised accumulation of inhibiting compounds, longer retention time and start-up period, reduced digester efficiency in methane yield and volatile solid (VS) reduction capacity^6^. Consequently, a continuous freshwater supply is important for the efficient AD of organic matter.

Water scarcity is a pressing issue both in India and globally. Evidence suggests that insufficient water is a significant barrier to the adoption of AD plants^7^. For instance, water scarcity resulted in 60% of biogas plants in Ethiopia being non-operational^8^ and hindered the implementation of household-level biogas plants in sub-Saharan Africa^9^. These challenges highlight the significance of identifying a suitable alternative to freshwater for AD.

Domestic sewage is a potential alternative to freshwater and could be used to prepare the substrate slurry. Despite implementing various government schemes and initiatives in India, including the National River Conservation Plan and the National Ganga River Basin Authority, sewage management faces challenges^10–12^. In addition, many households do not have access to sewage systems and instead rely on septic tanks or other on-site treatment methods. However, these methods are insufficient, resulting in the contamination of freshwater sources. By using this domestic sewage, we could potentially address the dependency on freshwater and treatment of sewage while also producing biogas through AD of organic matter.

Two aquatic weeds were selected as substrates for this study: *Eichhornia crassipes* (water hyacinth, WH) and *Hydrilla verticillata* (HV). WH and HV were selected for this study because of their ubiquitous nature and ability to thrive under adverse conditions. WH is a floating aquatic weed, whereas HV is a submerged plant that can survive in environments with less than 1% sunlight and 7% salinity^13,14^. These weeds were selected for this study because of their impact on the native ecosystem, prolific propagation, and abundance. Researchers have suggested pretreatments for the breakdown of lignin in WH for effective degradation. Barua and Kalamdhad (2017)^15^ identified thermal pretreatment using a hot-air oven at 90°C for 60 min provided the best pretreatment for WH to produce pretreated water hyacinth (PWH). Studies on AD of WH and HV using co-digestion and pretreatment techniques have shown that both substrates have substantial biogas yield potential^16,17^. Klomjek and Sarin (2022)^18^ conducted a co-digestion study of rice straw and swine wastewater. They explored the effects of swine wastewater nutrients on rice straw’s AD. However, they have not explored the effects of microbes from swine wastewater on AD.

This study explores the sustainable use of domestic sewage as an alternative to freshwater for substrate slurry preparation in AD, focusing on the role of the sewage microbial community on AD while reducing the dependency on freshwater sources. The main objective of this study was to understand the potential of sewage as a substitute for freshwater in AD. This involves investigating how the introduction of sewage affects the overall efficiency and output of AD. A biochemical methane potential (BMP) study was conducted on WH, PWH, and HV using different food-to-microbe (F/M) ratios. This study measured key parameters, such as VS, volatile fatty acids (VFA), soluble chemical oxygen digestion (sCOD), and daily methane production. These measurements provide insights into the efficiency of the AD process and the extent of organic matter breakdown. 16S-rRNA and heterotrophic plate count (HPC) analyses of the cow dung (CD) and sewage were performed to analyse the role of microbes from CD and sewage in assisting the AD process. This analysis sheds light on the microbial dynamics within the AD system when sewage is introduced. This study also focuses on understanding the degradation of lignin, a complex organic polymer that is resistant to degradation, which, if not degraded properly, could hinder the efficiency of the AD process. By substituting the freshwater utilised in AD with domestic sewage, the dependency on freshwater could be reduced, and the treatment and utilisation of sewage, which might have otherwise found its way into surface water bodies or land and contaminated them, could be achieved.

## Materials and methods

### Substrate and inoculum

The WH and HV substrates were collected from Deepor Beel, Southwest of Guwahati, India. Studies have demonstrated that hydrolysis can be accelerated by pretreating WH, and methane production can be enhanced. Thermal pretreatment using a hot-air oven was identified as an effective technique, and the optimal pretreatment condition for WH was heating the shredded WH at 90°C for 60 minutes^15^. In this study, PWH was prepared using collected fresh WH, which was first shredded and then thermally pretreated. Fresh CD, used as the inoculum source, was collected from Amingaon village near the IIT Guwahati campus. The sewage was freshly collected from the sump well of a sewage treatment plant inside the IIT Guwahati campus, India. Initial characterisation of CD, sewage, WH, and HV was carried out immediately after their collection using the procedure detailed in Sect.  “Analytical methods” and detailed in Table 1.

**Table 1:** Initial characterisation of untreated and hot-air oven pretreated WH, HV, cow dung, and sewage.

	pH	MC (%)	sCOD (mg/L)	VFA (mg/L)	TS (%)	VS(%TS)	Lignin (%)
CD	7.01 ± 0.54	76.27 ± 0.01	1050 ± 25	800 ± 10	23.72 ± 0.01	50.96 ± 1.67	–
Sewage	7.12 ± 0.50	99.9 ± 0.02	125 ± 25	200 ± 10	0.1 ± 0.02	84 ± 4.50	–
WH	6.03 ± 0.50	89.86 ± 0.73	983.33 ± 28.86	416.66 ± 14.43	10.14 ± 0.73	71.43 ± 0.20	18.63 ± 0.82
PWH	6.21 ± 0.40	87.31 ± 0.01	1408.33 ± 14.43	475 ± 25	12.69 ± 0.01	77.46 ± 0.01	–
HV	7.30 ± 0.30	86.87 ± 0.004	575 ± 50	425 ± 25	13.13 ± 0.004	66.6 ± 0.11	14.50 ± 0.51

### Experimental setup

**Fig. 1:**
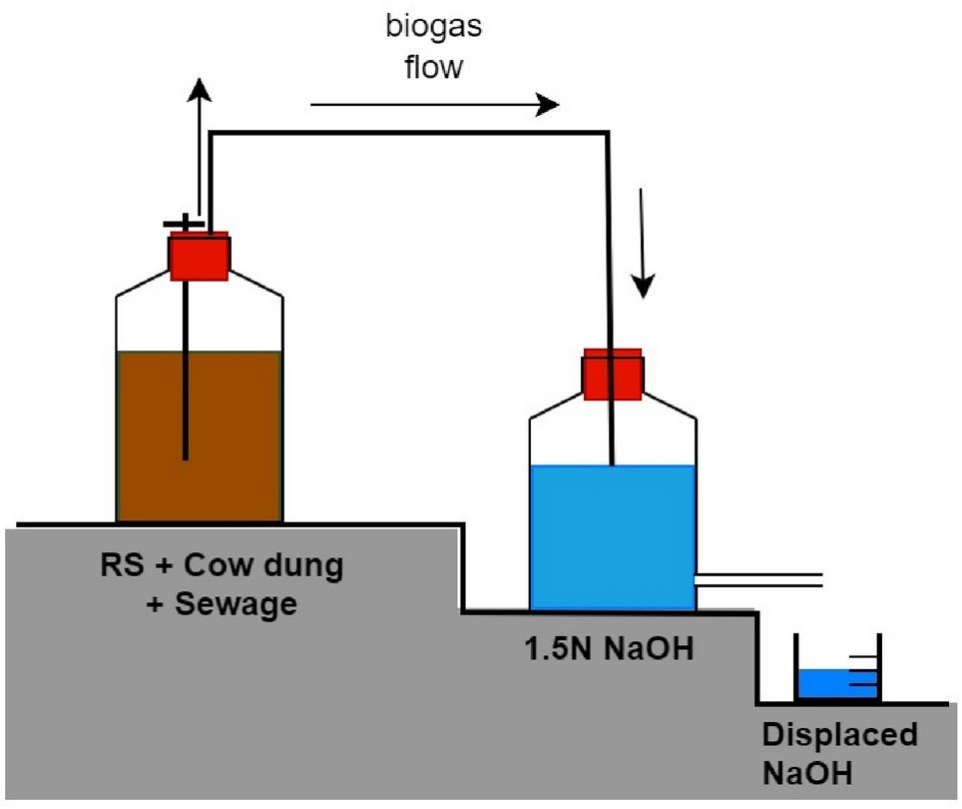
Schematic diagram of the Batch BMP setup.

The BMP setups for WH, PWH, and HV were studied (Fig. 1). The F/M ratios, varied from 0.5 to 2.5 with increments of 0.5, were studied in triplicates, and outliers in daily methane readings were eliminated using Dixon’s Q test^19^. The F/M ratios were calculated based on VS, where VS of the substrates was taken as food and that of CD was considered to be microbes^20^. BMP tests were conducted in batch mode in 1 L reagent bottles. The substrates were shredded and ground to a size smaller than 2 mm. Each reactor was fed with CD and substrates based on F/M ratio calculations, as shown in Table 2. The volume of each reactor was made up to 700 mL (70% of reactor volume) using domestic sewage, which acts as the medium. Control reactors for CD, substrates, and sewage were also set up and monitored in triplicates. The reactors were fed with small quantities of various diluted macronutrients (phosphate buffer) and micronutrients (Ferric Chloride, Calcium chloride, Magnesium Sulphate, Cobalt Nitrate, and Nickel Chloride) to facilitate microbial acclimatisation and then slowly purged with nitrogen for 2–3 min to ensure an anaerobic environment. The reactor was then sealed using butyl rubber corks and connected to an aspirator bottle containing 1.5N NaOH using a pipe. The biogas produced is measured using the liquid displacement method. Upon contact with NaOH, the CO_2_ in the biogas gets converted to Na_2_CO_3_ and is removed. The unreacted gas, which now mainly consists of CH_4_, pushes the liquid into the container below for collection and is measured daily. An alkali indicator (thymol blue) was used, and NaOH was replaced as soon as it was exhausted. Samples were collected from each reactor weekly, and parameters were checked to determine the progress of the AD process.

**Table 2:** Amount of substrate and cow dung taken for each BMP test setup.

F/M ratio	0.5	1.0	1.5	2.0	2.5
CD (g)	50	50	50	50	50
WH (g)	41.77	83.54	125.31	167.08	208.85
PWH (g)	34.84	69.69	104.54	139.38	174.23
HV (g)	34.55	69.09	103.64	138.19	172.74

### Analytical methods

The total solids (TS) were analysed by oven-drying the samples at 100 ± 5°C for 24 h and ignition at 550°C for 2 h in a muffle furnace to determine the VS. For sCOD and VFA, the samples were diluted 1:10 with distilled water and shaken at 150 rpm for 2 h before filtering. The filtered samples were used to analyse VFA and sCOD, whereas the unfiltered samples were used to determine the pH^21^. VFA were analysed using the pH titration method^22^. For VFA analysis, a 50 mL filtered sample was taken in a beaker, and the pH was adjusted to 3.3–3.5 using sulfuric acid. The sample was then lightly boiled using the open reflux method for 3 min and then cooled to room temperature while covering the mouth of the conical flask using aluminium foil. Titrate the cooled-down sample using 0.05N NaOH to pH 4, complete the titration to pH 7, and note the amount of NaOH used between pH 4 and 7. The volatile acid alkalinity (VAA) is calculated ($VAA = \frac{mL 0.05 N NaOH \times 2500}{{mL sample}}$). If the VAA is less than 180 mg/L, the VFA was reported the same as the VAA; if the VAA is greater than 180 mg/L, the VFA was reported as 1.5 times the value of VAA. For field emission scanning electron microscopy (FE-SEM) analysis and lignin degradation study, WH and HV samples were collected: fresh (before digestion) and digested separately in freshwater and sewage media. The FE-SEM and lignin analysis were carried out only for those F/M ratios with the highest methane yield. The samples were meticulously cleaned using running water and dried at 50°C in a hot-air oven until they were moisture-free. The samples were then thoroughly ground using a mortar and pestle and analysed at the central instrumentation facility of IIT Guwahati for FE-SEM imaging. The sample collected from freshwater and sewage media was also used to find the lignin degradation using the acid digestion method^23,24^.

HPC tests were performed to determine the number of heterotrophic bacteria present during the initial days of BMP^21^. Plate count agar was used for the study, and the spread plate method was adopted. 1 mL of fresh sewage and 1g of fresh CD were collected, mixed with sterile water in separate test tubes, and mixed thoroughly until a homogenous mixture was formed to create test samples of various dilutions. These diluted test samples were pipetted to petri dishes containing solidified and sterilised agar, then spread using a spreader and intubated for 5 days at 28°C. Plates containing 30–300 colonies were selected and counted on a colony counter before reporting. The sewage and CD samples were sent to external laboratories for 16S-rRNA analysis.

### Kinetic modelling and energy assessment

The cumulative methane yield was modelled using the modified Gompertz model (MGM), logistic function model (LFM), and transfer function model (TFM) to obtain the kinetic parameters that could be used to understand methane production.

1 $${\text{MGM}},\quad {\text{Y}} = {\text{M}} \times {\text{exp}}\left( { - {\text{exp}}\left[ {\frac{{{\text{Rm}} \times {\text{e}}}}{{\text{M}}}\left( {{\uplambda } - {\text{t}}} \right) + 1} \right]} \right)$$

2 $${\text{LFM}},\quad {\text{Y}} = \frac{{\text{M}}}{{1 + {\text{exp}}\left( {\frac{{4.{\text{Rm}} \times \left( {{\uplambda } - {\text{t}}} \right)}}{{\text{M}}} + 2} \right)}}$$

3 $${\text{TFM}},\;\;{\text{Y}} = {\text{M}}\left[ {1 - \exp \left( {\frac{{{\text{Rm}} \times \left( {{\uplambda } - {\text{t}}} \right)}}{{\text{M}}}} \right)} \right]$$

where Y is the cumulative methane production (mL) at time t (d), and M is the maximum methane production potential (mL). R_m_ (mL/d) is the maximum methane production rate, and λ is the lag phase time (d). These parameters were optimised using the ‘lsqcurvefit’ function in MATLAB (R2024a).

The energy potential of each AD is also calculated^25,26^. The energy output (E_out_, kJ/kg VS_added_) was calculated using Eq. 4, where Q is the cumulative methane in m^3^/kg VS_added_. The lower heating value (LHV) for methane is 35.8 MJ/m^3^ CH_4_, and ϕ_ε_ is the efficiency of the biogas generator, assumed to be 36% ^27^.

4 $${\text{Eout}} = {\text{Q}} \times {\text{LHV }} \times \phi {\upvarepsilon }$$

The energy used (E_in_, kJ/kg VS_added_) for pretreatment was calculated using Eq. 5, where P is the power of the equipment in watts (3000 W for hot air oven), t is the time in hours (1h, for this study), and w is the weight in terms of VS of the substrate treated in one cycle (1.77 kg for this study).

5 $${\text{Ein}} = \left( {{\text{P}} \times {\text{t }} \times 3.6} \right)/w$$

6 $${\text{Net energy}},{\text{ E }} = {\text{ E}}_{{{\text{out}}}} - {\text{ E}}_{{{\text{in}}}}$$

## Results and discussion

### 16S-rRNA and HPC

The HPC test showed that the reactors that used sewage had 10^2^–10^4^ times the heterotrophic bacterial count compared to those that used freshwater. Microbes belonging to the phyla *Bacteroidetes*, *Firmicutes*, *Planctomycetes*, and *Spirochaetes* facilitate hydrolysis^28–31^. *Proteobacteria*, however, contain microbes that predominantly help in hydrolysis and also help carry out acidogenesis and acetogenesis^28,29^. Actinobacteria then use the hydrolysis products of* Proteobacteria* to produce propionic acid^31–33^. Phylum *Euryarchaeota,* belonging to the kingdom Archaea, helps in methane production^34^. The 16S-rRNA results of sewage and CD are shown in Table 3. In sewage, nearly 85% of the microbial population consisted of hydrolytic bacteria, less than 7% were methanogenic archaea, and the remainder were microbes essential for acidogenesis and acetogenesis. CD was used as inoculum in this study, and its 16S-rRNA sequencing indicated that 45% of the microbes were hydrolytic bacteria, 25% methanogenic archaea, and the remainder were acidogenic and acetogenic bacteria. While microbes from sewage, which are predominantly hydrolytic bacteria, helped in speeding up the hydrolysis process of the hard-to-digest substrates such as WH and HV, the microbes from CD supplemented the microbes that were lacking in sewage for carrying out acidogenesis, acetogenesis and methanogenesis. Additionally, facultative microbes from sewage create a favourable environment for methanogens by consuming trace oxygen, fostering an optimal environment for the growth and activity of methanogens. While sewage alone cannot be used as inoculum because most of the microbes present are predominantly hydrolytic, these abundant hydrolytic bacteria contribute significantly to speeding up the substrate hydrolysis rate, as shown in subsequent sections.

**Table 3:** Microbial population distribution using 16S-rRNA sequencing in CD and domestic sewage.

Kingdom	Phylum	CD (%)	Sewage (%)
**Archaea**	*Euryarchaeota*	25	6
	Others	2	0.6
**Bacteria**	*Bacteroidetes*	19	30
	*Proteobacteria*	19	46
	*Planctomycetes*	9	–
	*Firmicutes*	8	9
	*Actinobacteria*	5	3
	*Spirochaetes*	4	0.8
	*Chloroflexi*	2	0.9
	Others	7	3.7

### Methane production

F/M ratio 2.0 yielded the highest methane yield of 4380 ± 28 mL (241.5 mL CH_4_/g VS_added_) for WH at the end of 50 days of digestion. Compared to the study conducted by Barua and Kalamdhad (2017)^35^, where freshwater was used as the medium and CD as inoculum, the use of sewage as the medium resulted in a nearly 50% increase in cumulative methane production for the AD of WH. More than 80% of the cumulative methane yield was observed to be produced within the first 35 days. This increase in cumulative methane production is attributed to the action of hydrolytic microbes from sewage, which helps in the early breakdown of lignin and enhances the microbial access of cellulose and hemicellulose. At the end of 50 days, an F/M ratio of 2.0 generated the maximum methane, followed by F/M ratios of 2.5, 1.5, 1.0, and 0.5.

Barua and Kalamdhad (2017)^35^ reported a cumulative methane production of 3039 ± 32 mL for BMP of PWH in 32 days when freshwater was used as a medium. In this study, where sewage was used as the medium, the AD of PWH was completed in 34 days, yielding 3635 ± 15 mL (200.5 mL CH_4_/g VS_added_) of methane, a 19.6% increase. However, the cumulative methane produced by PWH in the sewage medium was less than that produced by untreated WH in the sewage medium, possibly because of the sudden accumulation of VFA in PWH, leading to the inhibition of AD. In the case of PWH, the time taken to complete the AD was similar in both freshwater and sewage mediums, likely because the pretreatment process disrupts the complex structure of lignocellulosic biomass, making it accessible to the microbes. As a result, the additional hydrolytic microbes from sewage have a limited impact on the overall hydrolytic rate. Peak daily methane production (168 mL) was achieved by F/M 2.0 on day 6. The maximum cumulative methane was produced by F/M 2.0, followed by 2.5 > 1.5 > 1.0 > 0.5, respectively.

For the AD of HV in sewage medium, the entire digestion was completed in just 44 days, achieving a cumulative methane yield of 3853 ± 47 mL (212 mL CH_4_/g VS_added_) by F/M 2.0. In contrast, the study by Sathyan et al. (2023)^36^ reported a digestion period of 50 days and a methane yield of 157.69 mL CH_4_/g VS_added_ when using freshwater and CD as medium and inoculum, respectively, indicating that the use of sewage as medium significantly accelerated the AD of HV, reducing digestion period by 12% and increasing the methane yield by 35%. It can be observed that the F/M ratio of 2.0 has the highest methane production, followed by the F/M ratios of 2.5, 1.5, 1.0, and 0.5, respectively.

**Fig. 2:**
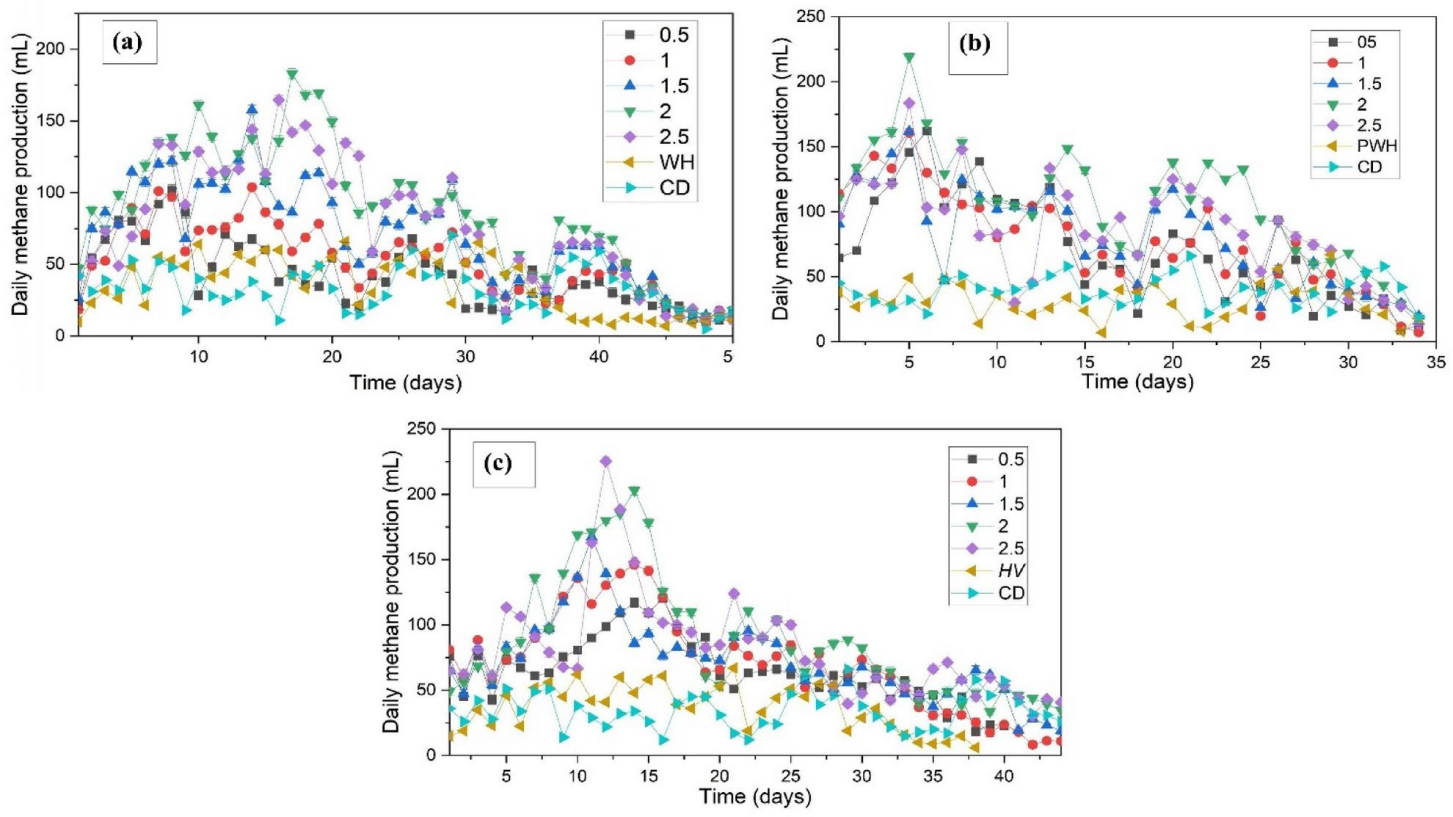
Daily methane production during AD of (**a**) WH (**b**) PWH, and (**c**) HV in sewage media.

**Fig. 3:**
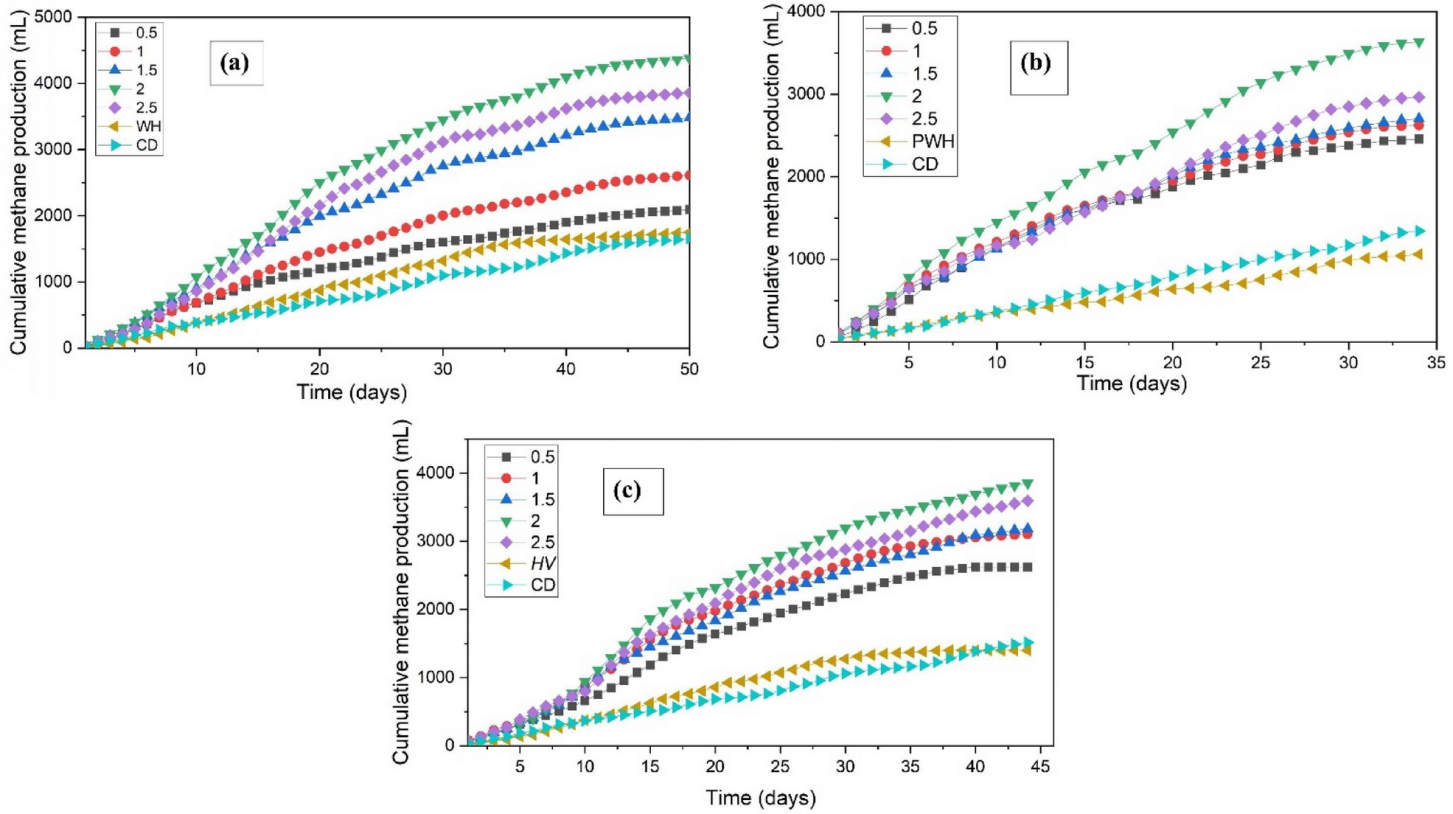
Cumulative methane production during AD of (**a**) WH (**b**) PWH, and (**c**) HV in sewage media.

The AD of WH, PWH, and HV in sewage medium resulted in substantial methane production. Digesters utilising sewage as the medium exhibited accelerated methane production compared to those using freshwater, suggesting that the use of sewage could eliminate the need for energy-intensive pretreatment steps, as the additional hydrolytic bacteria from sewage can effectively break down complex organic matter, similar to the effects of pretreatment. Hydrolytic and facultative bacteria from sewage, which created a favourable environment for methanogens, contributed to an earlier peak in methane production. This reduction in the overall digestion time positively affected AD efficiency. The daily and cumulative methane readings are shown in Figs. 2 and 3, respectively.

### Volatile solids

**Fig. 4:**
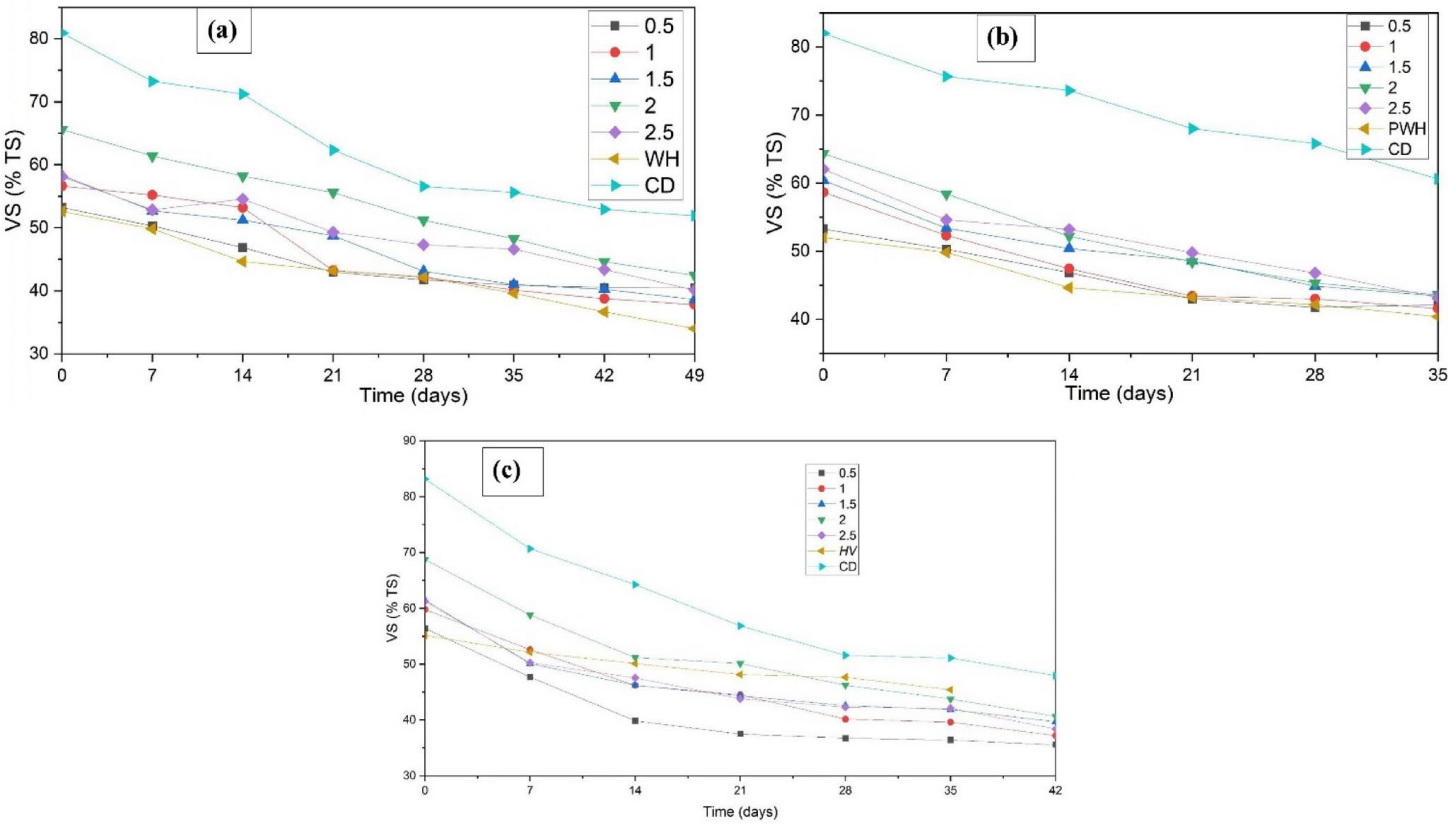
VS (%TS) concentration during AD of (**a**) WH (**b**) PWH, and (**c**) HV in sewage media.

VS has extensively been used in studies to assess the biodegradability of organic matter, and a direct relationship between the amount of VS reduced and the amount of biogas produced has been established^37^. A VS reduction of 35.3% in WH was observed at an F/M ratio of 2.0, the highest among all ratios. For the same ratio, the maximum methane production was achieved. A VS reduction of 32.21% was observed for F/M 2.0 in the case of PWH. This reduction was comparable to that observed in AD without pretreatment. Hence, using sewage media instead of pre-treatment results in similar degradation and saves considerable energy. Similarly, a VS reduction of 40.86% for HV was observed for F/M 2.0 (Fig. 4), which agrees with methane production at the same ratio.

### VFA and sCOD

**Fig. 5:**
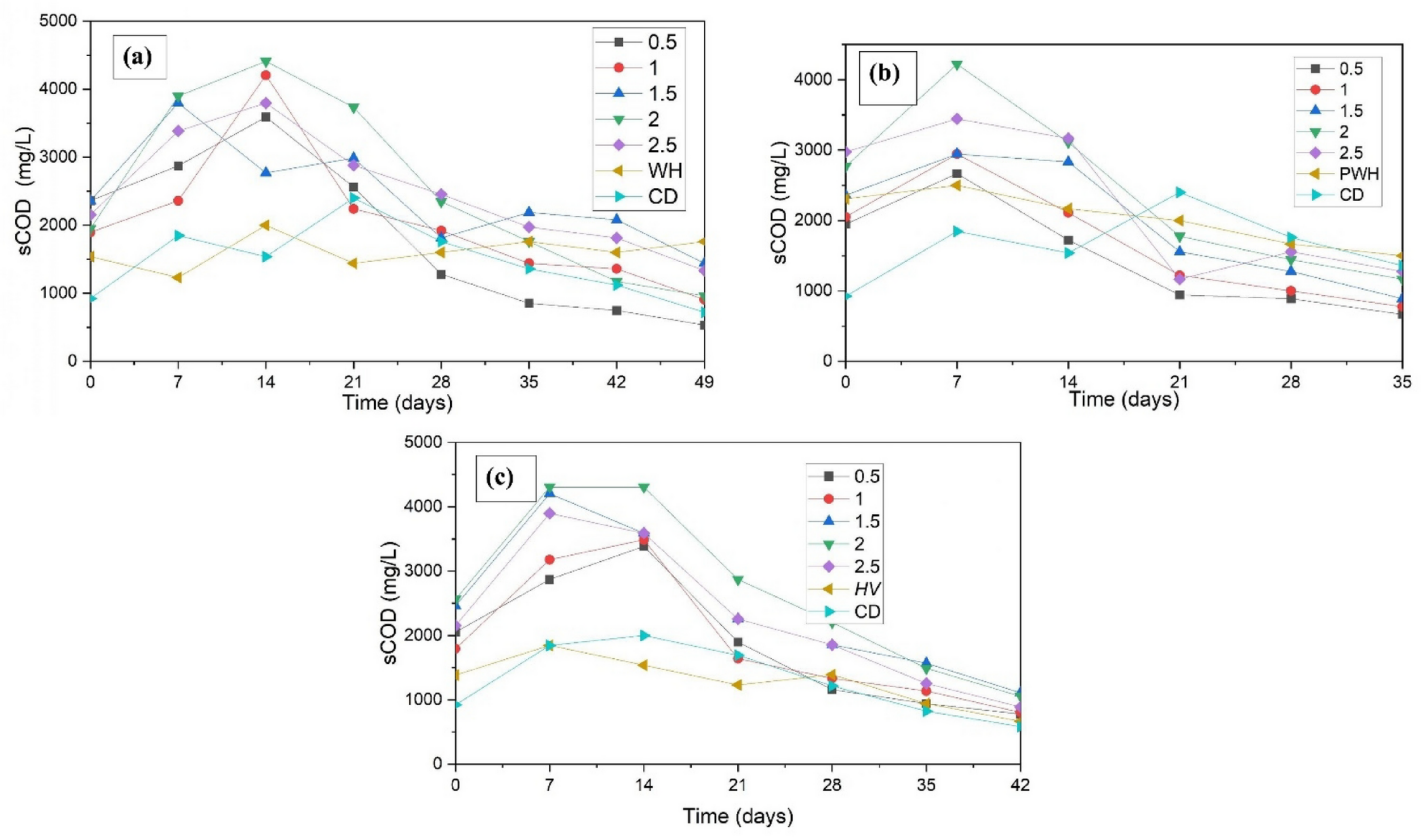
sCOD concentration during AD of (**a**) WH (**b**) P WH, and (**c**) HV in sewage media.

**Fig. 6:**
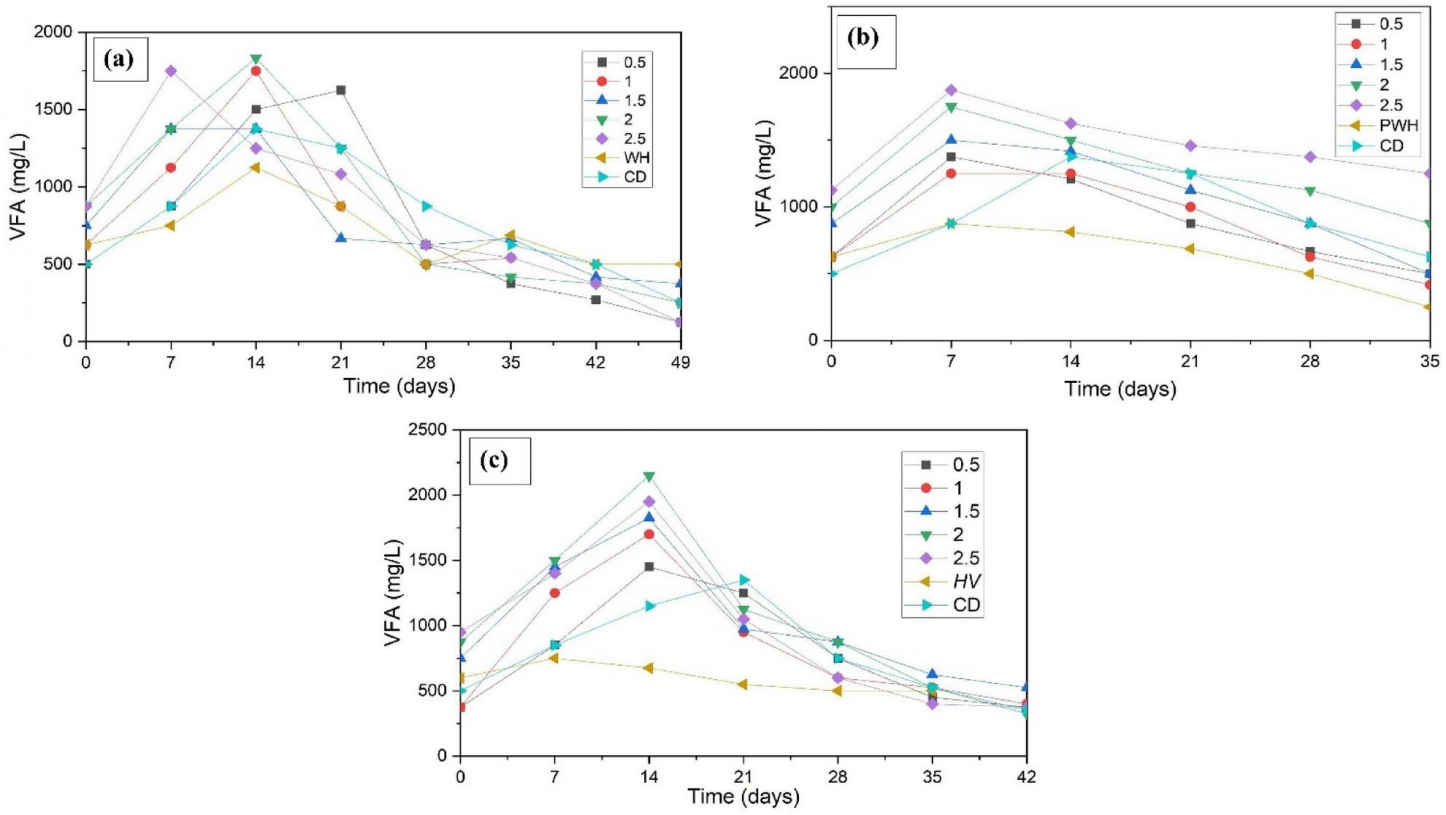
VFA concentration during AD of (**a**) WH (**b**) P WH, and (**c**) HV in sewage media.

Each of the four steps of AD, hydrolysis, acidogenesis, acetogenesis, and methanogenesis, occur concurrently, and one or more processes may be dominant at any one time. The dominant AD phase was identified using the trends in the sCOD and VFA (Figs. 5 and 6) at any given time. sCOD increases when the solubilisation rate of the substrate (during hydrolysis) is faster than the consumption rate of the solubilised product (during acidogenesis). When acidogenesis overtakes hydrolysis as the dominant process, the sCOD levels decrease. VFA levels increased when the VFA production rate (by acidogenesis) exceeded the consumption rate (by acetogenesis). It begins to decline when acetogenesis becomes more prominent than acidogenesis. Ammonia produced during AD acts as a buffer, neutralising the pH drop caused by VFA synthesis^38^.

The sCOD and VFA increased during the first two weeks of the untreated WH BMP test. After the first 2–3 weeks, both the sCOD and VFA levels decreased. F/M ratio 2 yielded the highest sCOD and VFA levels of 4410 mg/L and 1833 mg/l, respectively. During the BMP of PWH, an F/M ratio of 2 produced the highest sCOD of 4222 mg/L, followed by an F/M ratio of 2.5. However, the VFA for an F/M ratio 2.5 (1875 mg/L) was higher than that with an F/M ratio 2.0 (1750 mg/L). No discernible trends in VFA or sCOD were observed with respect to the methane production^15^.

During the AD study of HV, sCOD steadily increased until the 7^th^ day for all F/M ratios except F/M 2.0, after which it declined, whereas VFA production increased steadily until the 14^th^ day, after which it decreased. The highest VFA (2150 mg/L) and sCOD (4307 mg/L) concentrations were observed at the optimal F/M ratio 2.0. The sCOD and VFA values of WH and HV showed a considerable increase in the first 2 weeks of AD, indicating that the hydrolysis and the acidogenesis are progressing unhindered. In PWH’s case, the VFA increase occurred in the first week, affecting the methanogens and methane yield, as explained in Sect.  “Methane production”.

### Lignin degradation

Lignin is a biopolymer found in the cell walls of plants that poses a challenge to biodegradation due to its limited bioavailability^39^. Lignin analysis revealed a notable improvement in their degradation. WH and HV’s initial lignin content were 18.63 ± 0.82% and 14.5 ± 0.51%, respectively. In the case of WH, a degradation of 28.32% was observed in the sewage medium in just 14 days, whereas it took 21 days to achieve a similar degradation of 32.63% in the freshwater medium. HV also followed similar trends, where the sewage medium degraded 38.34% lignin in 14 days, of which 21.64% degradation was achieved within the first 7 days, whereas the freshwater medium degraded 28.29% in 14 days. This accelerated lignin degradation in the sewage medium provides evidence that the hydrolytic bacteria from the sewage significantly improved the lignin degradation rate in both WH and HV.

### FE-SEM analysis

**Fig. 7:**
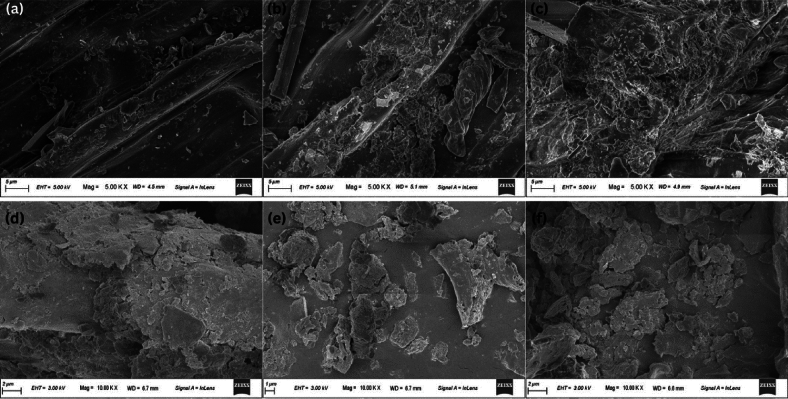
Morphological changes in WH and HV after AD. (**a**) Fresh, undigested WH; (**b**) WH after 1 week of AD with water; (**c**) WH after 1 week of AD with sewage. (**d**) Fresh, undigested HV; (**e**) HV after 1 week of AD with water; (**f**) HV after 1 week of AD with sewage.

Comparative examination of the FE-SEM images of the fresh WH and HV to those digested for one week in sewage and freshwater media revealed significant differences in morphology. The images showed a noticeably higher degree of substrate degradation when sewage was used than when freshwater was used, suggesting that sewage microbial communities actively helped break down plant cell walls (Fig. 7). Fresh WH and HV appeared relatively smooth in the FE-SEM images. In contrast, those digested with sewage showed distinct signs of degradation, with increased surface roughness, cracks, and potential disintegration compared to the freshwater-digested samples. These cracks improve the biomass availability and subsequently lead to better degradation. This observation aligns with the 16S-rRNA results and the lignin degradation results. In summary, FE-SEM analysis provides strong visual evidence that sewage media promotes higher breakdown of substrates.

### Kinetic modelling and energy assessment

**Fig. 8:**
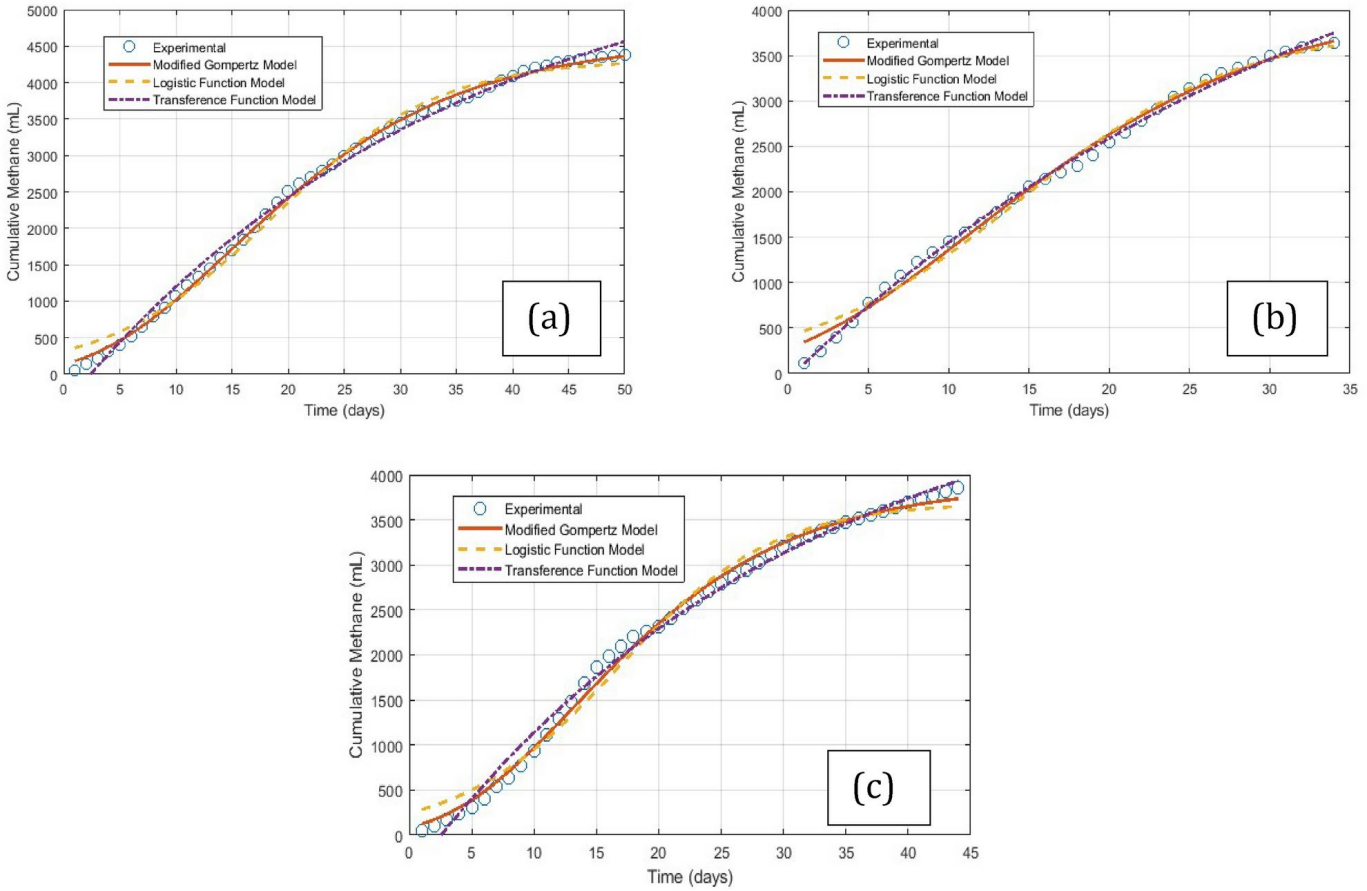
Model fit for F/M ratio (2.0) with highest cumulative methane yield (**a**) WH, (**b**) PWH, and (**c**) HV.

The MGM, LFM, and TFM models were analysed for all three BMP studies. The fitted graphs and their corresponding kinetic parameters for F/M ratios with the highest cumulative methane production are shown in Fig. 8. and Table 4. All three models exhibited high R^2^ values (> 0.9855), indicating a strong fit with the experimental data. λ is the point on the curve where the growth rate reaches its maximum and is called the lag phase. When λ is positive, there is an initial lag phase followed by an acceleration in growth. When λ is negative, lag occurs before the start of the process, which does not make sense in a physical sense in most kinetic models. For some MGM and LFM analyses, λ was found to be negative, and TFM was noted as the best model. The λ values for WH and HV were found to be less than 4 days, and for PWH, it was less than 1 day in most cases. These values are quite low for highly lignocellulosic WH and HV, indicating that the accelerated methane production started in the early stages of AD, further validating the claim that the sewage medium’s microbes help improve the hydrolysis rate. Although the λ values were very low for PWH, this was as expected due to pretreatment. The Rm values for the MGM of WH, PWH and HV were 143.54 mL/d, 136.25 mL/d and 144.93 mL/d, respectively. This value signifies the maximum methane production rate during the AD process. The substrate and inoculum quality, ambient conditions and presence of inhibiting compounds generally influence the Rm values. In this study, the high values of Rm are due to the combined action of microbes from sewage and CD, which accurately explains the accelerated hydrolysis and higher methane yields.

**Table 4:** Kinetic parameters from models for F/M ratio with highest cumulative methane production for each substrate.

Substrate	Model	F/M	Y	M	R_m_	λ	R^2^
WH	MGM	2	4362.10	4585.91	143.545	3.0420	0.9986
	LFM	2	4261.19	4322.30	146.587	3.9664	0.9926
	RFM	2	4563.13	6135.60	175.485	2.3984	0.9945
PWH	MGM	2	3661.98	4191.84	136.257	0.0273	0.9940
	LFM	2	3607.47	3812.16	139.576	0.7522	0.9884
	RFM	2	3752.07	6281.45	169.857	0.3612	0.9976
HV	MGM	2	3735.37	3908.78	144.930	3.3886	0.9961
	LFM	2	3649.52	3696.85	146.955	4.0949	0.9874
	RFM	2	3931.71	5340.56	171.694	2.5506	0.9924

An energy assessment of methane produced for the AD of WH, PWH, and HV in a sewage medium was carried out. The results showed that the net energy production for WH was 4664 J/g VS_added,_ and for HV, it was 4109 J/g VS_added_. Although PWH has shown substantial methane yield in the sewage medium, the net energy value was -2684 J/g VS_added_ owing to the significant energy demand for pretreatment. These findings highlight the importance of considering energy consumption for pretreatment while evaluating the overall efficiency of the AD process. The energy potential from AD of WH and HV indicates that using sewage as a medium is much more efficient than using costly pretreatments.

### Practical implications and limitation

Using sewage as a substitute for freshwater in AD has multiple benefits, such as reduced dependency on freshwater, treatment of sewage that would otherwise be difficult to deal with, and significant methane yield. Due to sewage’s low organic content, sewage control setups produced insufficient methane. The microbes in sewage are predominantly hydrolytic and help accelerate the hydrolysis, eliminating the need for pretreatment. However, the lack of microbes essential for acidogenesis, acetogenesis and methanogenesis makes it dependent on additional inoculum sources, such as CD, as elaborated by the 16S-rRNA test. While sewage media provides multiple benefits to lignocellulosic substrates, further studies are required to fully understand the nature of the digestate produced at the end of digestion.

## Conclusion

The study demonstrates the potential of substituting freshwater with sewage as the medium in AD of organic waste. The results indicate that the sewage medium enhances the hydrolysis rate and has significant methane yield. The lignin degradation study, FE-SEM imaging and kinetic study show an accelerated rate of hydrolysis in sewage medium compared to the freshwater medium. The energy assessment study showed that using sewage could avoid the necessity of pretreatment. Substituting freshwater with sewage conserves water and utilises readily available waste biomass for sustainable methane production. Further research with diverse substrates and large-scale systems and analysis of the digestate produced are recommended to validate these findings and optimise the field application process.

## Supplementary Information

Supplementary Information.

## Data Availability

The data supporting this study’s findings are available from the corresponding author upon reasonable request.
