# Supplementary material for: Domestic sewage as a sustainable freshwater substitute for enhanced anaerobic digestion of lignocellulosic biomass
Source: Sci Rep. 2024 Dec 30;14:31964. doi: 10.1038/s41598-024-83546-6 (PMC11685457; doi:10.1038/s41598-024-83546-6)
Supplement: Supplementary file 1 — Supplementary Information. [file 41598_2024_83546_MOESM1_ESM.docx]

Supplementary table 1: Kinetic parameters from models for different F/M ratios of each substrate

| **Substrate** | **Model** | **F/M** | **Y** | **M** | **R_m_** | **λ** | **R^2^** |
| --- | --- | --- | --- | --- | --- | --- | --- |
| WH | MGM | 0.5 | 2049.6 | 2202.84 | 58.2287 | -0.5197 | 0.9905 |
|  |  | 1 | 2567.26 | 2745.09 | 77.3195 | 1.6249 | 0.9955 |
|  |  | 1.5 | 3448.28 | 3646.01 | 108.622 | 2 | 0.9969 |
|  |  | 2 | 4362.1 | 4585.91 | 143.545 | 3.042 | 0.9986 |
|  |  | 2.5 | 3839.35 | 4000.73 | 133.946 | 3.9559 | 0.9991 |
|  | LFM | 0.5 | 2017.84 | 2076.11 | 57.4022 | -0.1345 | 0.9809 |
|  |  | 1 | 2516.21 | 2574.15 | 78.0171 | 2.3966 | 0.9871 |
|  |  | 1.5 | 3376.06 | 3436.08 | 109.689 | 2.7788 | 0.9895 |
|  |  | 2 | 4261.19 | 4322.3 | 146.587 | 3.9664 | 0.9926 |
|  |  | 2.5 | 3745.25 | 3784.82 | 137.498 | 4.926 | 0.9937 |
|  | TFM | 0.5 | 2105.61 | 2724.71 | 82.0683 | 0.8017 | 0.9985 |
|  |  | 1 | 2663.04 | 3632.22 | 99.566 | 1.8039 | 0.9986 |
|  |  | 1.5 | 3586.33 | 4741.38 | 139.423 | 1.9756 | 0.9974 |
|  |  | 2 | 4563.13 | 6135.6 | 175.485 | 2.3984 | 0.9945 |
|  |  | 2.5 | 4039.86 | 5392.31 | 158.09 | 2.8252 | 0.9914 |
| PWH | MGM | 0.5 | 2407.5 | 2518.68 | 113.527 | 0.5515 | 0.9921 |
|  |  | 1 | 2598.86 | 2809.79 | 104.902 | -0.9897 | 0.9924 |
|  |  | 1.5 | 2694.24 | 2943.62 | 109.087 | 0.0019 | 0.9963 |
|  |  | 2 | 3661.98 | 4191.84 | 136.257 | 0.0273 | 0.994 |
|  |  | 2.5 | 3013.73 | 3592.19 | 106.494 | 0 | 0.9939 |
|  | LFM | 0.5 | 2365.62 | 2402.99 | 111.058 | 0.7434 | 0.9815 |
|  |  | 1 | 2562.34 | 2646.75 | 102.88 | -0.8145 | 0.9841 |
|  |  | 1.5 | 2648.07 | 2737.79 | 110.137 | 0.5359 | 0.991 |
|  |  | 2 | 3607.47 | 3812.16 | 139.576 | 0.7522 | 0.9884 |
|  |  | 2.5 | 2968.74 | 3189.48 | 110.917 | 0.9392 | 0.99 |
|  | TFM | 0.5 | 2488.81 | 3017.41 | 159.986 | 1.1466 | 0.9977 |
|  |  | 1 | 2660.69 | 3444.56 | 150.63 | 0.1486 | 0.9988 |
|  |  | 1.5 | 2769.88 | 3897.91 | 144.319 | 0.5097 | 0.9981 |
|  |  | 2 | 3752.07 | 6281.45 | 169.857 | 0.3612 | 0.9976 |
|  |  | 2.5 | 3082.48 | 6249.88 | 125.059 | 0.0342 | 0.9962 |
| HV | MGM | 0.5 | 2663 | 2835.68 | 95.569 | 2.8728 | 0.9988 |
|  |  | 1 | 3102.61 | 3245.83 | 118.201 | 2.5947 | 0.9984 |
|  |  | 1.5 | 3118.46 | 3362.78 | 106.127 | 2.2113 | 0.9962 |
|  |  | 2 | 3735.37 | 3908.78 | 144.93 | 3.3886 | 0.9961 |
|  |  | 2.5 | 3483.28 | 3710.27 | 124.786 | 2.8401 | 0.9969 |
|  | LFM | 0.5 | 2601.24 | 2650.26 | 98.4121 | 3.7955 | 0.9945 |
|  |  | 1 | 3040.79 | 3083.91 | 118.061 | 3.1466 | 0.9924 |
|  |  | 1.5 | 3053.41 | 3131.81 | 108.182 | 3.0206 | 0.9882 |
|  |  | 2 | 3649.52 | 3696.85 | 146.955 | 4.0949 | 0.9874 |
|  |  | 2.5 | 3399.69 | 3464.32 | 128.167 | 3.7074 | 0.9899 |
|  | TFM | 0.5 | 2786.34 | 4080.96 | 111.543 | 1.9948 | 0.9917 |
|  |  | 1 | 3246.12 | 4260.26 | 145.747 | 2.0458 | 0.9926 |
|  |  | 1.5 | 3248.47 | 4811.5 | 128.402 | 1.8668 | 0.997 |
|  |  | 2 | 3931.71 | 5340.56 | 171.694 | 2.5506 | 0.9924 |
|  |  | 2.5 | 3649.42 | 5333.56 | 146.769 | 2.1087 | 0.994 |

| 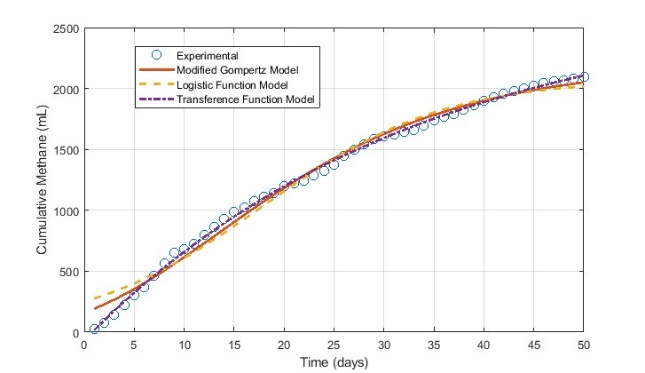 | 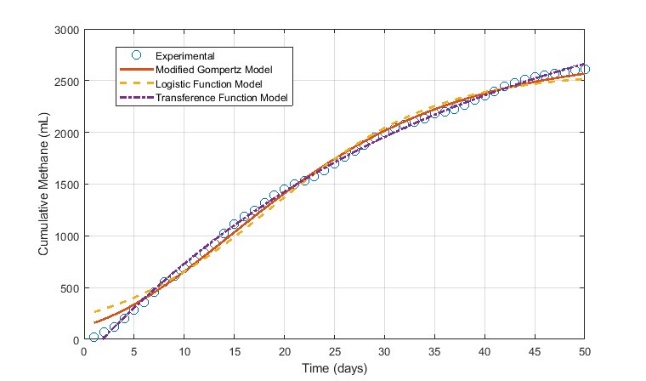  b |
| --- | --- |
| 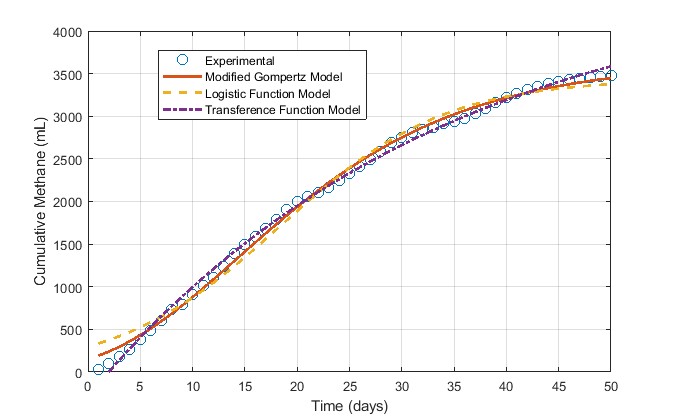  c | 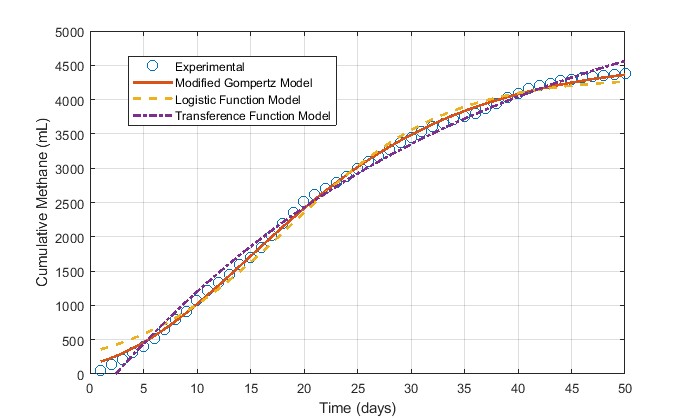  d  a |
| 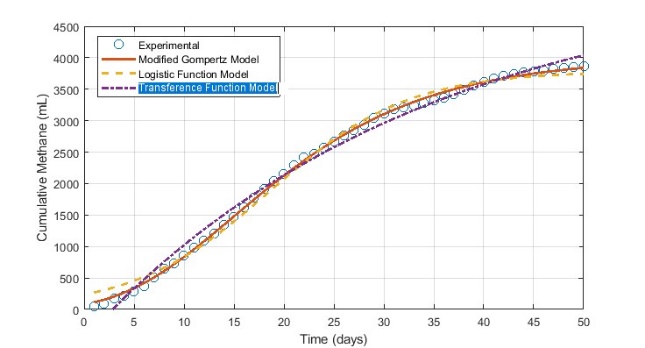  e |  |
| Supplementary fig 1: Model fit for cumulative methane yield of WH for F/M ratios a) 0.5 b) 1.0 c) 1.5 d) 2.0 and e) 2.5 | |

| 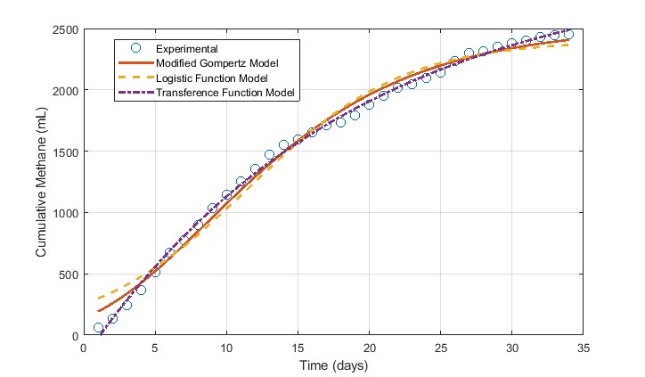 | 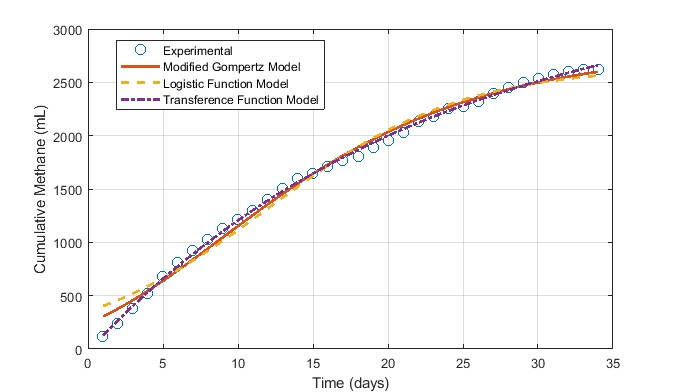  b |
| --- | --- |
| 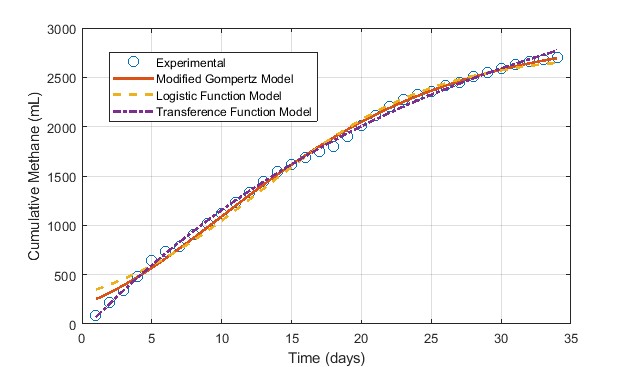  c | 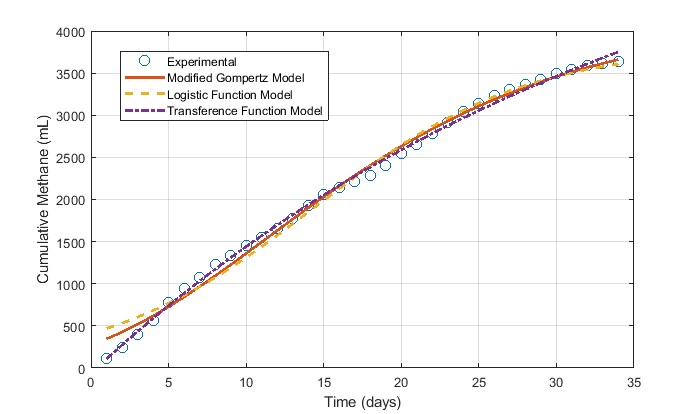  d  a |
| 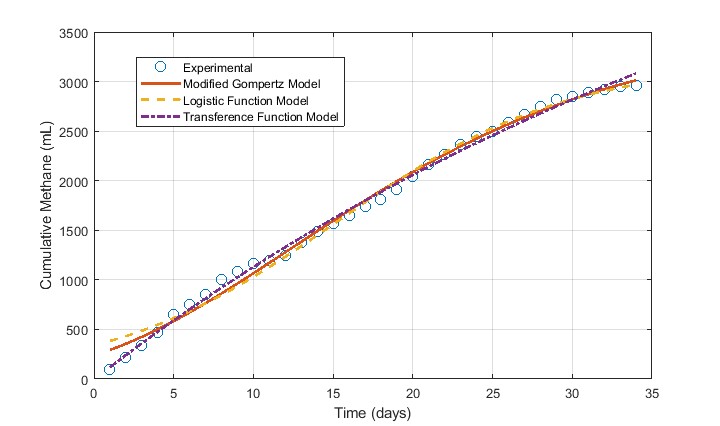  e |  |
| Supplementary fig 2: Model fit for cumulative methane yield of PWH for F/M ratios a) 0.5 b) 1.0 c) 1.5 d) 2.0 and e) 2.5 | |

| 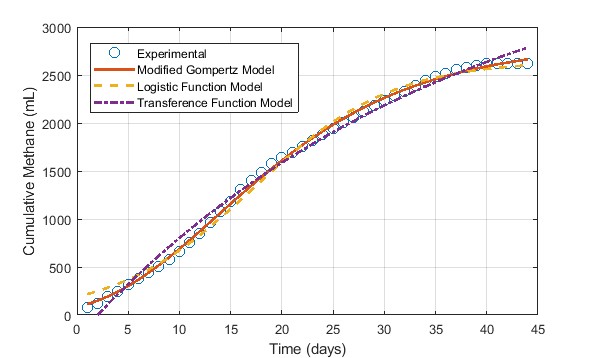 | 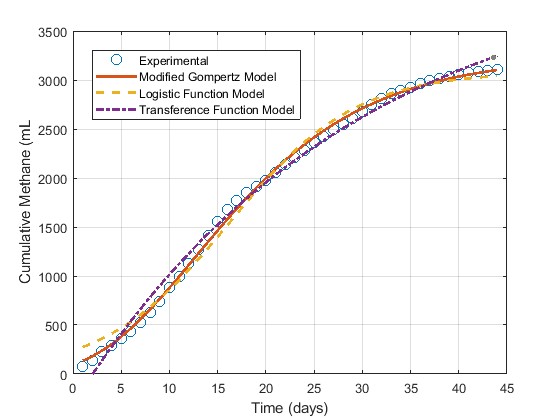  b |
| --- | --- |
| 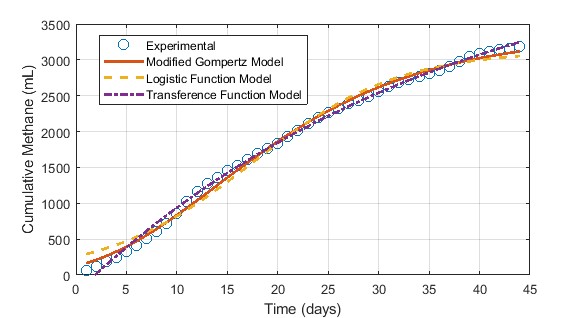  c | 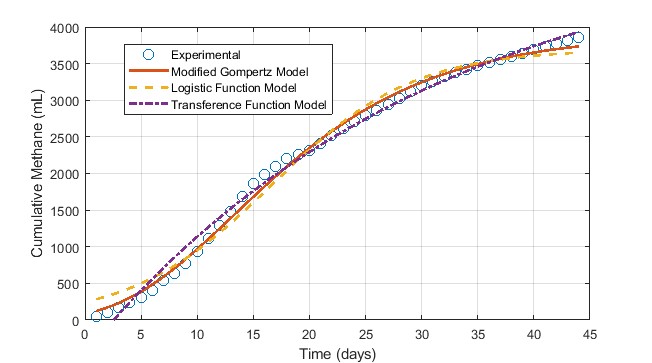  d  a |
| 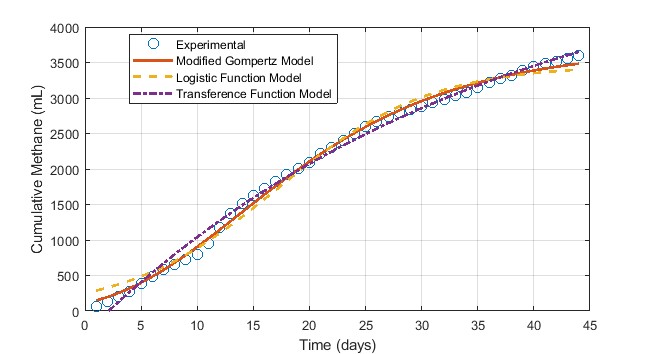  e |  |
| Supplementary fig 3: Model fit for cumulative methane yield of HV for F/M ratios a) 0.5 b) 1.0 c) 1.5 d) 2.0 and e) 2.5 | |
